# Ulinastatin Alleviates Repetitive Ketamine Exposure-Evoked Cognitive Impairment in Adolescent Mice

**DOI:** 10.1155/2022/6168284

**Published:** 2022-12-12

**Authors:** Yu Hong, Shiyu Meng, Shouping Wang, Ting Liu, Jiayi Liu

**Affiliations:** ^1^Department of Anesthesiology, Sun Yat-sen Memorial Hospital, Sun Yat-sen University, Guangzhou, China; ^2^The Third Affiliated Hospital of Guangzhou Medical University, Guangzhou, China

## Abstract

Ketamine (KET) is widely used for induction and maintenance of anesthesia, and long-term use is required for treatment of depression patients. Repeated use of KET is associated with mood and memory disorders. Ulinastatin (UTI), a urinary trypsin inhibitor, has been widely undertaken as an anti-inflammatory drug and proved to have neuroprotective effects. The aim of this work was to determine whether prophylactic use of UTI could attenuate KET-induced cognitive impairment. It was found that repetitive KET anesthesia cause cognitive and emotional disorders in adolescent mice in WMZ and OFT test, while UTI pretreatment reversed the poor performance compared to the AK group, and the platform finding time and center crossing time were obviously short in the CK+UTI group (*P* < 0.05). Our ELISA experiment results discovered that UTI pretreatment reduced the expression levels of IL-1*β* and IL-6 induced by CK anesthesia compared to AK (*P* < 0.05). In addition, UTI pretreatment protected the cognitive function by restraining the expression levels of Tau protein, Tau phospho-396 protein, and A*β* protein in the CK group compared to the AK group in Western blotting (*P* < 0.05). The results suggested that UTI could act as a new strategy to prevent the neurotoxicity of KET, revealing a significant neuroprotective effect of UTI.

## 1. Introduction

Annually, millions of young patients undergo surgeries and receive general anesthetic. With the rapid development of modern medical technology, pediatric surgery is becoming more complicated and even requiring multiple surgeries. In addition, the corresponding anesthesia is becoming more frequent with longer time.

However, many animal studies have raised questions regarding the safety of pediatric anesthesia and its effects on normal brain development. Thus, it got widespread attention in many healthcare professionals including international anesthesia community, the neuroscientist community, and the electronic media [1]. This is considered as a huge challenge in health setup and needs to be addressed on priority basis.

Perioperative neurocognitive dysfunction (PND) is accompanied by clinical manifestations in learning and memory, where impairment of attention is one of the most common postoperative complications associated with high mortality. General anesthetics can impair the neurocognitive function by inducing abnormal aggregation of *β*-amyloid peptide (A*β*), neuroinflammation, Tau hyperphosphorylation, and cell apoptosis [1]. It is reported that general anesthetics may evoke cognitive disorders and emotional disorders such as depression and anxiety [2].

Ketamine (KET) is widely used clinically with strong analgesic effects and slight respiratory depression. It is usually utilized in pediatric patients for basic general anesthesia for dressing changes and check-ups requiring sedation and immobilization [3, 4]. Although many mechanisms of KET such as the abnormal aggregation of A*β* [3, 5, 6], the overexpression and phosphorylation of Tau protein, and neuroinflammation have been proposed, the exact mechanisms are still unclear [7]. Neuroinflammation is closely related to PND [8]. Our previous study found that anti-inflammatory measures could reduce PND [5]. However, investigations have rarely concentrated on the influences of repetitive KET exposure on neurodegeneration. Hence, this work is aimed at exploring the influences of repetitive KET administration on learning and memory abilities and emotional abilities in the animal model.

UTI is a multivalent Kunitz-type serine protease inhibitor, which is also called the urinary trypsin inhibitor. It is synthesized from an intertrypsin inhibitor through proteolytic cleavage by neutrophil elastase at the site of inflammation. Besides, UTI is also widely known as an anti-inflammatory drug acting on various organs, and it shows numerous pharmacological effects such as neuroprotective effects. And it can alleviate the production of inflammatory cytokines such as TNF-*α*, IL-1, IL-6, and CRP, which may be attributed partly to the suppression of NF-*κ*B pathway and mitogen-activated protein kinases (MAPKs) [5]. In addition, UTI is able to inhibit various serine proteases such as trypsin, chymotrypsin, neutrophil elastase, and plasmin. Due to these characteristics, UTI is used in anti-inflammatory therapy in encephalomyelitis of mice. Currently, few research has been reported on the learning and emotional impairments caused by prolonged ketamine exposure. Besides, whether ulinastatin can improve ketamine-induced postoperative cognitive dysfunction and its mechanism are still unclear. Our work is aimed at evaluating UTI as a new therapeutic drug for its neuroprotective effect [6, 9].

## 2. Materials and Methods

### 2.1. Animal Groups and Treatment

21-day-old specific pathogen free (SPF) male C57BL/6 pups (weight: 20~30 grams) were selected in this work. Pups were assigned to five groups: acute treatment control group (AC group), acute KET treatment group (AK group), chronic treatment control group (CC group), chronic KET repetitive treatment group (CK group), and chronic KET combined with UTI pretreatment group (CK+UTI group).

Each pup in the CK group was injected with KET at the same dose of 30 mg/kg intraperitoneally, 3 times a day, 30 minutes apart, for 7 consecutive days. The dosage of ketamine was referenced from literature [10]. The mice in the AK group received the same treatment but only for 1 day, while those in the CK+UTI group were injected with UTI (50,000 U/kg) intraperitoneally 30 minutes before the first KET injection daily. In the AC and CC groups, mice were not exposed to any drugs [10].

**Figure 1:**
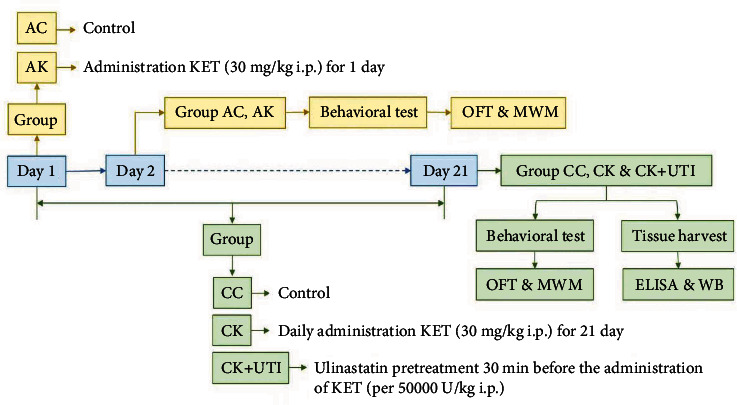
A brief schematic representation of experimental protocols.

12 mice were used for neurocognitive tests including open field test (OFT) and Morris water maze (MWM), which were carried out 24 hours after the last injection of KET. Six mice were decapitated at the 24th hour after the last KET administration to harvest the fresh hippocampus of juvenile mice. Enzyme-linked immunosorbent assay (ELISA) method was applied to analyze the immune factors. Meanwhile, expressions of dementia-related proteins were detected by Western blotting. The experimental timeline was shown in Figure 1.

### 2.2. MWM

The cognitive function of pups in each group was evaluated by using the MWM test, which included two parts: navigation and exploration. The navigation experiment was in the first three days, where the platform was removed and the free trajectories of the pups were observed on the last day. All experimental methods refer to the literature [11]. The trajectories were tracked by video and the consequences were analyzed by EthoVision XT 7.0.

### 2.3. OFT

12 mice were gently placed in a transparent plastic box, and the pups were free to explore for 15 min; all experimental methods refer to the literature [12]. Movements were observed by video and the consequences were analyzed using EthoVision XT 7.0.

### 2.4. ELISA

The expressions of interleukin (IL)-1*β*, IL-6, and tumor necrosis factor *α* (TNF-*α*) in the hippocampal extractives were measured using the ELISA (Rapid Bio-Lab, West Hills, CA). Hippocampus homogenate was obtained from the three groups and centrifuged at 10,000 g for 15 min at 4°C to collect the supernatant. ELISA was then employed to measure the hippocampal levels of the proinflammatory cytokines, IL-1*β*, IL-6, and TNF-*α* according to the instructions from manufactures. All ELISA results were obtained by measuring the optical density (OD) values at 450 nm, and the mean values were calculated from at least three experiments.

### 2.5. Western Blotting

The protein sample from centrifugal supernatant of mice hippocampus was heated and denatured with 1/5 volume of 5X electrophoresis sample buffer, and the sample was loaded at 40 *μ*g/well. After electrophoresis with 10% sodium dodecyl sulfate and polyacrylamide gel, the protein was transferred onto a polyvinylidene fluoride (PVDF) membrane. After sealing with 5% skim milk, the primary antibody (1 : 2,000 rabbit anti-Tau antibody, 1 : 2000 rabbit anti-A *β* antibody, and 1 : 2,000 mouse anti-*β*-actin antibody) was added for incubation. After overnight at 4°C, a corresponding secondary antibody was added for washing and incubation of the blots. Diaminobenzidine (DAB) chromogenic agent, darkroom pressing, film scanning, and Image-Pro Plus (IPP) system were adopted to calculate the intensity of the interested protein.

### 2.6. Statistical Analysis

Results were shown as mean ± standard deviation (SD) and analyzed by Solutions-Statistical Package for The Social Sciences (SPSS) 22.0. The groups were statistically compared by using the one-way analysis of variance (ANOVA) and least significant difference (LSD) test. *P* value < 0.05 meant as the difference was statistical significance.

## 3. Results

**Figure 2:**
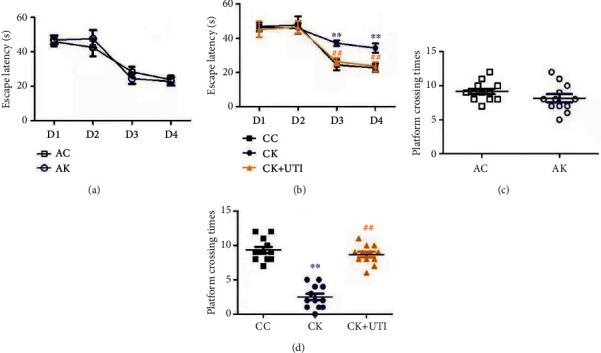
Repetitive KET-induced learning and memory impairment. (a) and (b) demonstrated the sailing time; (c) and (d) showed the platform-crossing times. All consequences were expressed as mean ± SD (*n* = 12). ^∗∗^*P* < 0.01, and ^##^P < 0.01.

In MWM, the escape latency in the CK group was obviously longer compared with that in the CC group (Figure 2(b)). On the last two days, the performance between the AC and AK groups was nondifferentiated (Figure 2(a)). In the exploration parts, poor scores were clearly demonstrated in the CK group compared with the CC group (Figure 2(d)) and showed no obvious difference between groups AC and AK (Figure 2(c)). Compared with the CK group, group CK+UTI showed a shorter escape latency and increased platform-crossing times (Figures 2(b) and 2(d)). It was found that repetitive KET exposure in mice was more likely to evoke the learning and memory impairment, which can be mitigated by UTI.

**Figure 3:**
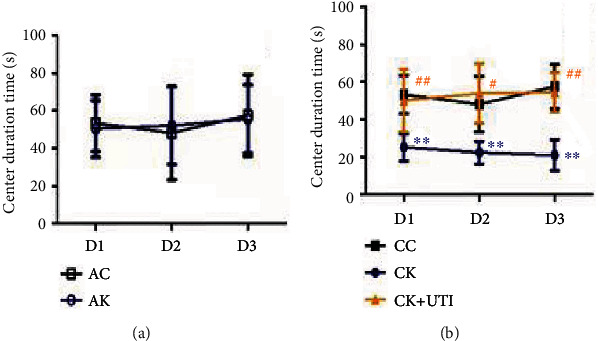
Repetitive KET treatment in mice evoked anxiety-like behaviors. (a) and (b) showed the center duration time. All result data were expressed as mean ± SD (*n* = 12). ^#^*P* < 0.05, ^∗∗^*P* < 0.01, and ^##^*P* < 0.01.

The OFT test showed that compared with the CC group, the duration time at the center was shorter in the CK group (Figure 3(b)), and the performance between the AC and AK groups was nondifferentiated (Figure 3(a)). Compared with the CK group, the CK+UTI group demonstrated less anxiety-like behaviors (Figure 3(b)), indicating that repetitive KET treatment was more likely to induce the anxiety-like behavior and which could be relieved by UTI pretreatment.

**Figure 4:**
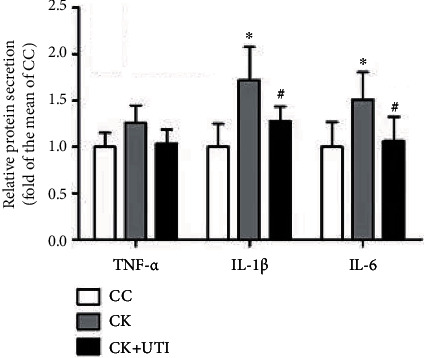
Repetitive KET-induced neuroinflammation can be alleviated by UTI. All result data were reported as mean ± SD (*n* = 6). ^∗^ and ^#^ meant *P* < 0.05 and *P* < 0.01, respectively.

The expressions of IL-1*β* and IL-6 in the CK group increased significantly, but that of TNF-*α* showed no obvious difference. UTI pretreatment reduced the expressions of IL-1*β* and IL-6 as contrasted with the CK group (Figure 4).

**Figure 5:**
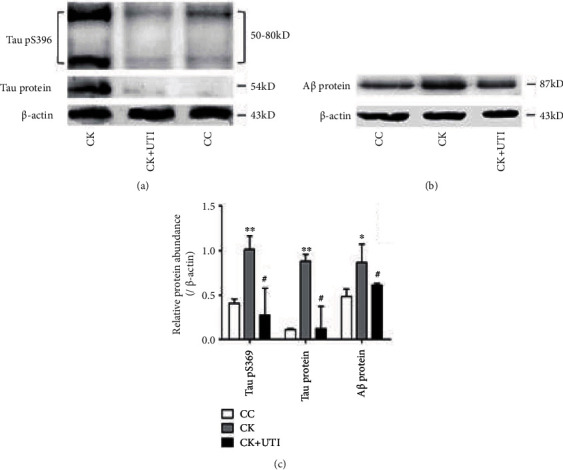
(a) and (b) showed the Western blotting results of Tau pS396, Tau protein, and A*β* protein. (c) illustrated the statistical value of each protein. ^∗^*P* < 0.05, ^∗∗^*P* < 0.01, and ^#^*P* < 0.01.

The expressions of Tau protein, Tau phospho-396 protein, and A*β* protein in the hippocampus were measured. Consistently, the expressions of Tau protein, Tau phospho-396 protein, and A*β* protein in the CK group were significantly upregulated compared with the CC group. In addition, the expressions of the above three proteins were all decreased after the UTI pretreatment compared with the CK group (Figure 5).

## 4. Discussion

KET is a widely used general anesthetic in young patients, for its efficacy of sedation and analgesia [13, 14]. However, KET is abused by many teenagers worldwide due to lapses in regulation and supervision. Hence, it should address exploring the causes of KET-evoked central nervous impairment in young individuals and prevention of long-term cognitive impairment [4]. Although numerous studies have shown significant neurodegeneration and neurological impairment due to KET anesthesia [15–19], rarely researcher has concentrated on the influences of repetitive anesthesia on cognitive disorders in adolescent mice. This work reported the preclinical evidence that short KET exposure in adolescent mice was not associated with memory impairment or emotional disorders, but the repetitive KET treatment was.

Previous studies have reported the adversaries associated with KET-like neuroapoptosis—neuronal cell death, typically when neonatal rodents and nonhuman primates received high doses and/or repetitive administration [15, 16, 19–23]. However, only few studies focus on adolescent animals so far with repetitive KET administration. Animals during adolescence are prone to impairment of central nervous system upon exposure to any adverse external environmental stimuli. It has been verified that high doses and frequent use of KET could cause brain disorders in adolescent rats and accelerate the neuronal apoptosis and that mice repetitively exposed to KET during adolescence showed extensive impairment [24, 25]. To better imitate the actual clinical use, the acute and repetitive KET treatment in a nontoxic dose were compared and found that PND occurred in repetitive KET exposure in contrast to the acute single administration.

A typical clinical manifestation of PND is learning and memory impairment [1]. Interestingly, significant anxiety-like behavioral disorders and memory impairment were found after repetitive KET treatment, as shown in Figure 3 [15, 26]. It was confirmed that teenagers who underwent repeated anesthesia and surgery during infancy were more likely to develop anxiety-like behaviors later [27, 28]. Similar results have been reported in clinical studies; exposure to anesthesia in a kid's early life would have bad affections in key brain development steps leading to behavioral and emotional disorders later in life [29, 30]. All of this suggests that KET should be applied carefully in teenagers and that alternatives should be sought when repeated application of KET is required.

In the MWM test, compared with the rats in the CK group, the rats in the CK+UTI group showed a reduction in the escape latency and increased time spent in the target quadrant, indicating that learning and memory impairment was alleviated for mice in the CK+UTI group. It was speculated that the improved cognitive function was related to the anti-inflammatory action of UTI. Compared with the CK group, the mice in the CK+UTI group showed increased time spent in the target quadrant and the increased frequency of crossing the platform. This indicated that the ability of mice to learn and memorize in the CK+UTI group was improved more significantly. More inflammatory factors were observed in the CK group in ELISA analysis, so it was speculated that UTI facilitated the beneficial effect on learning and memory impairment by inhibiting the cytokine production and reducing the inflammatory reaction in the hippocampus.

Neuroinflammation is an inflammatory response of the central nervous system (CNS) mediated by abnormal secretion of proinflammatory cytokines, chemokines, and reactive oxygen/nitrogen species. It can lead to an ongoing pathologic process in the CNS by infiltration of peripheral immune cells, edema, neuronal atrophy, damage, and death overtime, which affect the neuronal structure and function during prolonged neuroinflammation, resulting in cognitive and functional impairments [31]. The findings in this work showed that the repetitive KET-exposed model elevated the expression levels of IL-6 and IL-1*β* in the hippocampus. Such findings are consistent with the results of other researches [21, 25, 32]. Compared with the CK group, UTI inhibited the expression levels of IL-6 and IL-1*β* in the hippocampus.

The CNS can produce a variety of cytokines involved in regulating the brain function. Cognitive impairment is associated with excessive release of inflammatory factors. IL-6 and IL-1*β* are typical proinflammatory cytokines. Studies have shown that IL-1*β* participates in CNS immune regulation, stimulates the proliferation of astrocytes and microglia, promotes neuronal development, mediates the synaptic plasticity, and plays an important role in learning and memory [1, 8].

The abnormal expression levels of A*β* and Tau proteins are closely related to the pathogenesis of Alzheimer's disease (AD). Tau phosphorylation, aggregation, and spread contribute to AD pathogenesis [7, 33]. Tau exerts an important role in stabilizing the microtubule system, and its excessive phosphorylation can lead to neuronal degeneration, thereby leading to the occurrence of cognitive dysfunction. A*β* induces the release of inflammatory mediators, promotes the neurotoxic injury, and causes vascular endothelial dysfunction, thereby aggravating the injury of cerebral nerves and vessels. These studies suggest a molecular pathogenic mechanism for impaired cognition after anesthesia [5, 34].

UTI is a 67 kDa glycoprotein purified from the urine of healthy humans, and it is a nonspecific protease inhibitor and a urinary trypsin inhibitor. In addition, it can treat acute inflammatory disorders, sepsis, toxic shock, and hemorrhagic shock [6]. It is reported that UTI can alleviate the cerebral ischemia-reperfusion injury by regulating inflammation and oxidative stress [35]. Another study also reported that UTI could attenuate the brain edema after intracranial hemorrhage (ICH) of male Sprague-Dawley rats and that the preliminary molecular mechanism may be related to the decrease of the expression levels of proinflammatory cytokines including IL-1*β* and TNF-*α* [36]. As the pharmacological action of UTI is more complex, including anti-inflammatory, immune regulation, and organ protection, the mechanism of neuroprotection of UTI preventing cognitive impairment is multiple and complicated.

In conclusion, the results in this work suggested that UTI could decrease the release of IL-1*β* and IL-6 inflammatory factors and alleviate the expression levels of Tau-pS396, Tau, and A*β* proteins in the hippocampus, thereby alleviating the learning and memory impairment in rats with repetitive KET exposure. Such findings indicate that the UTI pretreatment might be a promising pave to treat cognitive deficits after repetitive KET exposure.

## Data Availability

All data used to support the findings of this study are available from the corresponding author upon request.
